# Comprehensive transcriptomic analysis revealing the regulatory dynamics and networks of the pituitary-testis axis in sheep across developmental stages

**DOI:** 10.3389/fvets.2024.1367730

**Published:** 2024-02-19

**Authors:** Shanglai Li, Bingru Zhao, Hua Yang, Keke Dai, Yu Cai, Hui Xu, Peiyong Chen, Feng Wang, Yanli Zhang

**Affiliations:** Jiangsu Livestock Embryo Engineering Laboratory, Nanjing Agricultural University, Nanjing, China

**Keywords:** RNA-seq, pituitary-testis axis, sheep, regulatory network, developmental stages

## Abstract

Spermatogenesis is a complex process intricately regulated by the hypothalamic-pituitary-testis (HPT) axis. However, research on the regulatory factors governing the HPT axis remains limited. This study addresses this gap by conducting a comprehensive analysis of transcriptomes from the pituitary and testis tissues across various developmental stages, encompassing embryonic day (E120), neonatal period (P0), pre-puberty (P90), and post-puberty day (P270). Utilizing edgeR and WGCNA, we identified stage-specific genes in both the pituitary and testis throughout the four developmental stages. Notably, 380, 242, 34, and 479 stage-specific genes were identified in the pituitary, while 886, 297, 201, and 3,678 genes were identified in the testis. Subsequent analyses unveiled associations between these stage-specific genes and crucial pathways such as the cAMP signaling pathway, GnRH secretion, and male gamete generation. Furthermore, leveraging single-cell data from the pituitary and testis, we identified some signaling pathways involving BMP, HGF, IGF, and TGF-β, highlighting mutual regulation between the pituitary and testis at different developmental stages. This study sheds light on the pivotal role of the pituitary-testis axis in the reproductive process of sheep across four distinct developmental stages. Additionally, it delves into the intricate regulatory networks governing reproduction, offering novel insights into the dynamics of the pituitary-testis axis within the reproductive system.

## 1 Introduction

In male animals, the intricate process of spermatogenesis involves the orchestrated action of cell-specific gene products and collaboration among different cells. This process is under the strict governance of developmental regulations, which profoundly influence male reproductive health (1). The testis, serving as the primary site of sperm production, undergoes distinct developmental stages in male animals, including the embryonic period, neonatal phase, prepubertal stage, and sexual maturity. During embryonic development, primordial germ cells differentiate into supporting cells, steroidogenic cells, and germ cells within the developing testes. In the neonatal and prepubertal stages, spermatogenesis remains dormant, and no initiation of sperm production occurs. The production of spermatozoa commences at sexual maturity, typically around 6–9 months of age. The hypothalamic-pituitary-gonadal (HPG) axis is a precise regulatory system that regulates the development of the reproductive system (2). The hypothalamic-pituitary-testicular (HPT) axis is a critical component in the process of sperm production. The pituitary gland plays a crucial role in gonadotropin secretion (3), which secretes luteinizing hormone (LH) and follicle-stimulating hormone (FSH). These hormones are essential for maintaining normal spermatogenic function by synergistically regulating the development of spermatogenic cells, Leydig cells, and Sertoli cells in the testis, as well as the synthesis and secretion of gonadal steroid hormones. Besides, the research found that suppression of PRL increases serum concentrations of LH and testosterone in goats (4). The development and function of the testis are thus intricately linked to the regulatory mechanisms of the pituitary gland.

Recently, genomic studies on the pituitary-testis axis have revealed candidate genes and pathways that are potentially pivotal in regulating reproductive traits in animals. For instance, transcriptomic sequencing was used to identify the important role of GnRH and cAMP signaling pathways in rams with different sexual behaviors (5). Fifteen genes involved in neuroactive ligand-receptor and WNT signaling pathways have been identified as potentially crucial for the development of goose external genitalia (6). A study has demonstrated the expression patterns of lincRNAs during postnatal testicular development of goats (7). However, few studies focused on the interconnected mechanisms controlling the development of the pituitary and testis, particularly in male sheep. This gap highlights the need for comprehensive research to elucidate the coordinated regulation of the pituitary-testis axis, thereby advancing our understanding of male reproductive health in animals.

Hu sheep, renowned for their exceptional prolificacy in China (8), serve as an ideal model for investigating fecundity mechanisms. To elucidate the novel genes and regulatory networks of the pituitary-testis axis during various developmental stages in Hu sheep, we performed a comprehensive analysis of transcriptomes from both the pituitary and testis tissues by RNA sequencing (RNA-seq). This study encompassed four critical stages: embryonic development, neonatal period, pre-puberty, and post-puberty. We aimed to provide new insights into the functioning of the pituitary-testis axis across these diverse developmental stages. Our study identified key regulators and signaling pathways within these developmental stages of the testis so as to advance our understanding of spermatogenesis at the molecular level. The findings of this study not only enhance our comprehension of the intricate processes involved in male reproductive health, but also contribute to the identification of candidate genes crucial for male reproductive capability in Hu sheep.

## 2 Materials and methods

### 2.1 Sample collection and preparation

#### 2.1.1 Animal and tissue collection

All the experimental animals were obtained from Jiangsu Qianbao Sheep Industry Limited Company, Yancheng, China. The developmental period of the embryo lamb can be estimated based on the mating records of the ewe. The embryos (E120) were collected in pregnant ewes at 120 embryonic days (*n* = 5). In the other three stages, three replicates were collected based on the growth of lambs (*n* = 3). The pituitary and each right testis from each ram were collected and snap-frozen in liquid nitrogen immediately for RNA extraction.

#### 2.1.2 RNA isolation and qualification

Total RNA was extracted using the TRIzol method (Invitrogen, CA, USA) and treated with RNase-free DNase I (Takara, Kusatsu, Japan). RNA degradation and contamination was monitored on 1% agarose gels. RNA concentration and purity was measured using NanoDrop spectrophotometer (Thermo Scientific, DE, USA). RNA integrity was assessed using the Agilent 2100 Bioanalyzer (Agilent Technologies, CA, USA).

#### 2.1.3 Library preparation for transcriptome sequencing

RNA purification, reverse transcription, library construction and sequencing were performed at Beijing Allwegene Technology Company Limited (Beijing, China) according to the manufacturer's instructions (Illumina, San Diego, CA).

### 2.2 Data analysis

#### 2.2.1 Quality control

Raw data (raw reads) of fastq format were firstly processed through in-house perl scripts. In this step, clean data (clean reads) were obtained by removing reads containing adapter, reads containing ploy-N and low quality reads from raw data. At the same time, Q20, Q30, GC-content and sequence duplication level of the clean data were calculated. All the downstream analyses were based on clean data with high quality.

#### 2.2.2 Mapping analysis

The adaptor sequences and low-quality sequence reads were removed from the data sets. Raw sequences were transformed into clean reads after data processing. These clean reads were then mapped to the reference genome sequence (Ovis_aries, ARS-UI_Ramb_V2.0) by STAR (9). Only reads with a perfect match or one mismatch were further analyzed and annotated based on the reference genome.

#### 2.2.3 Identification of stage-specific genes

Stage-specific genes (FDR < 0.05) for the pituitary and testis were identified between one stage and others using the R package edgeR (10) and Mfuzz (11). All functional enrichment analyses were conducted for each stage-specific gene type using the R package clusterProfile (12).

#### 2.2.4 Weighted gene co-expression network analysis (WGCNA)

To investigate the co-expression patterns of genes in the pituitary and testis tissues across four distinct developmental stages, we used the R package WGCNA (13) to construct gene co-expression networks. The analysis involved the construction of a gene co-expression network based on pairwise correlations between gene expression profiles. To achieve this, a soft-thresholding power was selected to emphasize strong correlations and mitigate weaker ones. Modules of highly correlated genes were identified, and the eigengene of each module was calculated to represent the overall expression pattern of the module. Module-trait associations were then assessed to link co-expression modules with specific developmental stages. This comprehensive WGCNA approach facilitated the exploration of dynamic gene expression patterns and potential regulatory modules governing the developmental transitions in both pituitary and testis tissues. The sequence motif enrichment analysis of promoters of genes in hub modules was conducted by MEME v.5.5.4 (14), based on the JASPAR(2020) core non-redundant vertebrate motifs from Tomtom (15).

#### 2.2.5 Exploration of pituitary and testis cell–cell interaction patterns

Testis single-cell RNA sequencing data for Hu sheep were downloaded from the GEO database (GSE184343), while the pituitary data originated from our unpublished dataset. These two datasets were amalgamated for analysis, and the R package “Harmony (16)” was employed to eliminate batch effects. Utilizing marker genes, the clusters were delineated into 21 distinct clusters, with eight clusters representing pituitary cells and 13 clusters representing testicular cells. Subsequently, we utilized CellChat (17) to explore the intercellular communication dynamics between the pituitary and testicular cells.

## 3 Results

### 3.1 Characterization of transcriptional profiles in sheep pituitary gland and testis development

**Figure 1:**
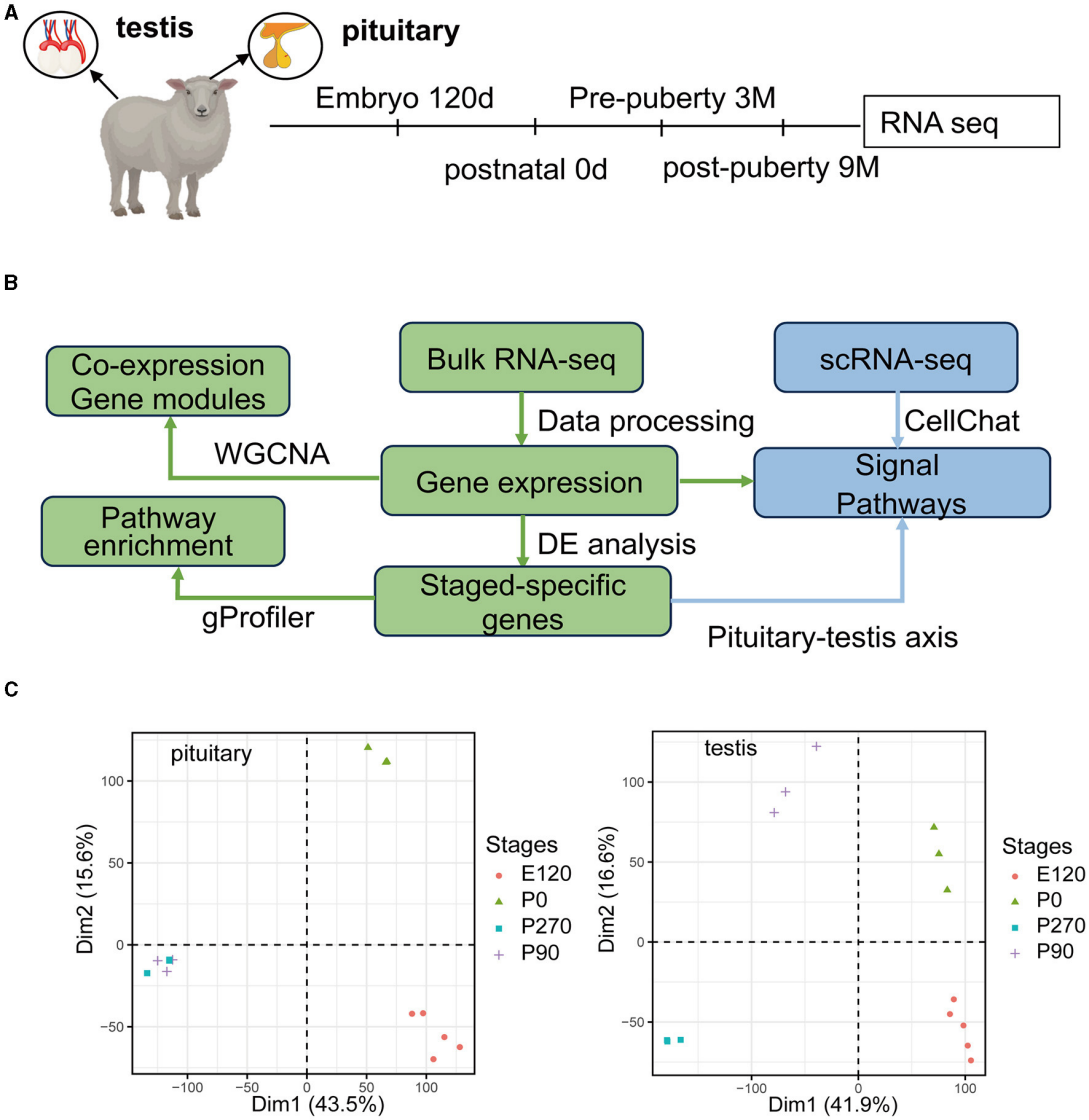
Overview of the pituitary and testis transcriptome during four development stages. **(A)** Schematic of experimental design. Marks on the timeline denote the age in which the pituitary gland and testis were collected. **(B)** Schematic of the analysis workflow. **(C)** PCA plot for pituitary gland and testis samples based on gene expression. After filtering for low-count genes, log_2_(normCounts+1) was used to perform principal component analysis (PCA).

**Figure 2:**
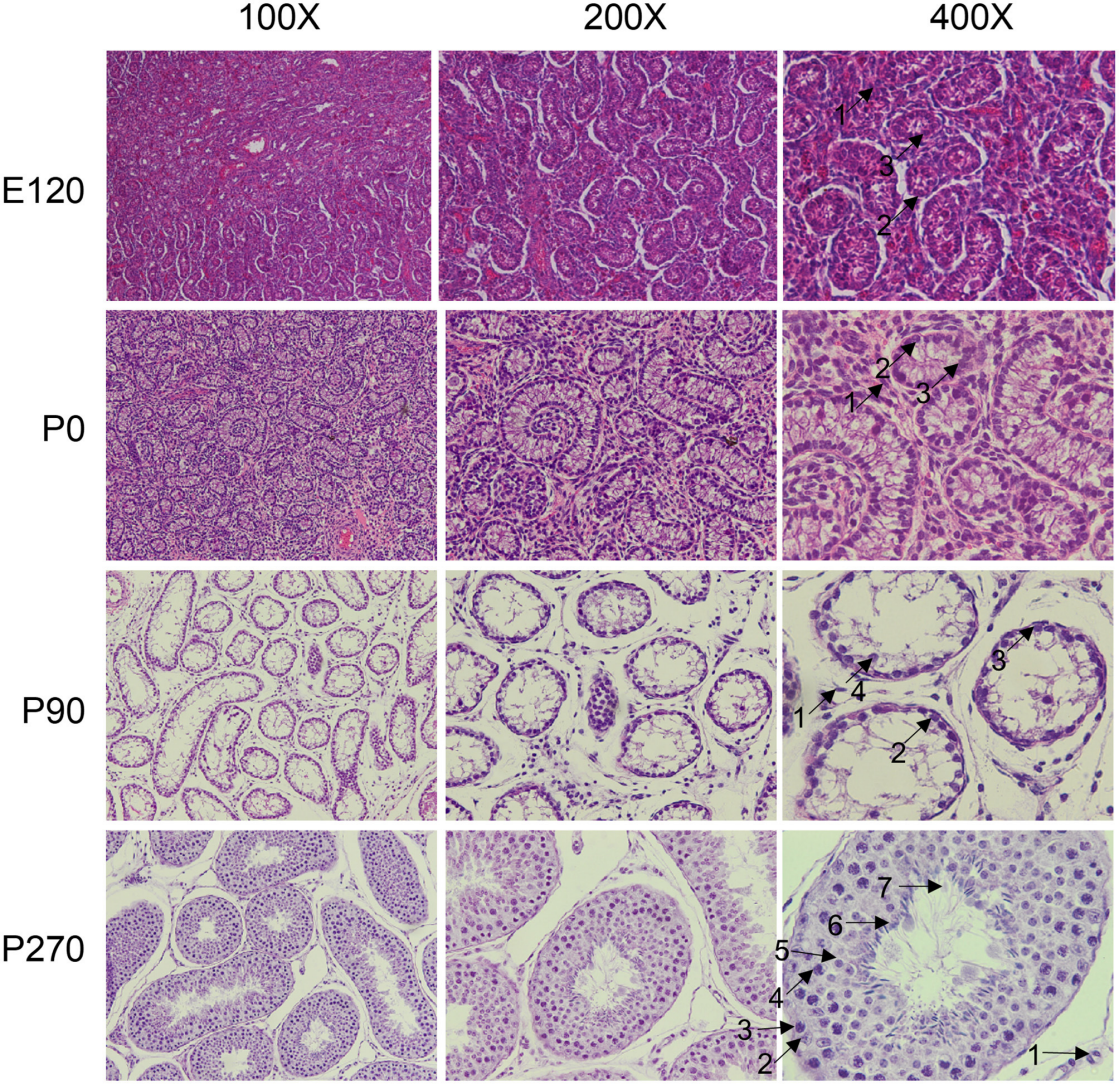
Histological analysis of testis at different developmental stage. The testicular tissues were observed under the microscope at × 100, × 200, and × 400 magnification. The different cell types were indicated by black arrows and numbered accordingly. 1: Leydig cells, 2: Sertoli cells, 3: spermatogonia, 4: spermatocytes, 5: round spermatids, 6: elongated spermatids, 7: sperm.

We collected pituitary and testis tissues from embryos (E120), newborns (P0) pre-puberty (P90), and post-puberty (P270) for transcriptome sequencing (Figure 1A). In total, we integrated 28 distinct RNA sequencing datasets across four developmental stages in pituitary and testis. Additionally, we integrated two single-cell sequencing datasets for analysis, a summary of the analysis workflow referred to Figure 1B. The developmental stage was consistently the main factor that distinguished the 14 samples based on their molecular profiles in the principal component analysis (PCA). Furthermore, we examined the histologic morphology of the pituitary gland and testis. In the testis of P0 and E120 groups, there were only a few Sertoli cells (Sg), Leydig cells (LCs), and spermatogonia cells (SCs). Spermatocytes appeared in the P90 stage, and fully developed sperm were observed at the P270 stage (Figure 2). There were no significant histological changes observed in the pituitary gland (Supplementary Figure S1).

### 3.2 Stage-specific genes in pituitary and testis across developmental stages

**Figure 3:**
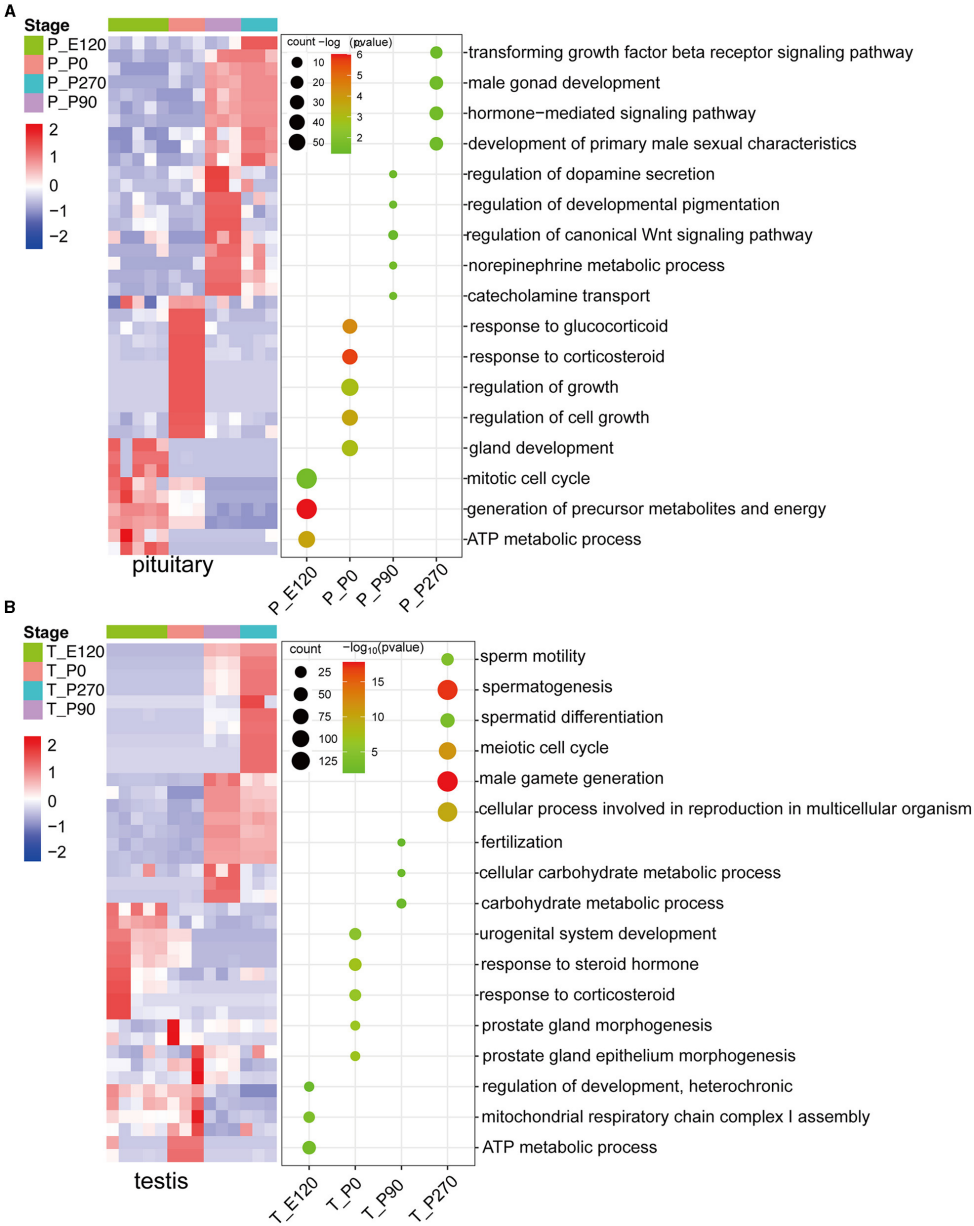
The upregulated stage-specific genes across developmental stages pituitary and testis. **(A)** Heatmap **(left)** shows the expression level [log_2_(TPM+ 1)] of the top 10 upregulated stage-specific genes across pituitary developmental stages; heatmaps **(right)** show the normalized consensus scores of significantly (FDR < 0.05) enriched Gene Ontology (GO) terms for all upregulated genes at each stage. The horizontal axis represents the various stages of the pituitary. **(B)** Similar to **(A)**, but for the testis.

To investigate key genes in the pituitary and testis across various developmental stages in Hu sheep, we conducted a detailed analysis of the upregulated genes at each stage and subsequently performed GO enrichment analysis on these genes. Our results demonstrated that during the embryonic and neonatal stages of pituitary development, the upregulated genes were significantly enriched in the mitotic cell cycle and ATP metabolic processes. As age progressed, there was a notable shift in the pituitary gene expression profile, with enrichment of processes related to hormone secretion, growth regulation, and male sexual characteristics (Figure 3A).

In a parallel analysis of testis development, we observed that the upregulated genes during the embryonic and neonatal stages were primarily enriched in energy metabolism and the regulation of cellular metabolic processes, including ATP synthesis. Notably, upon reaching sexual maturity, a significant upregulation of genes in the testis were predominantly associated with reproductive processes such as fertilization and male gamete generation (Figure 3B).

### 3.3 Dynamic expression patterns of genes during the development of pituitary and testis

**Figure 4:**
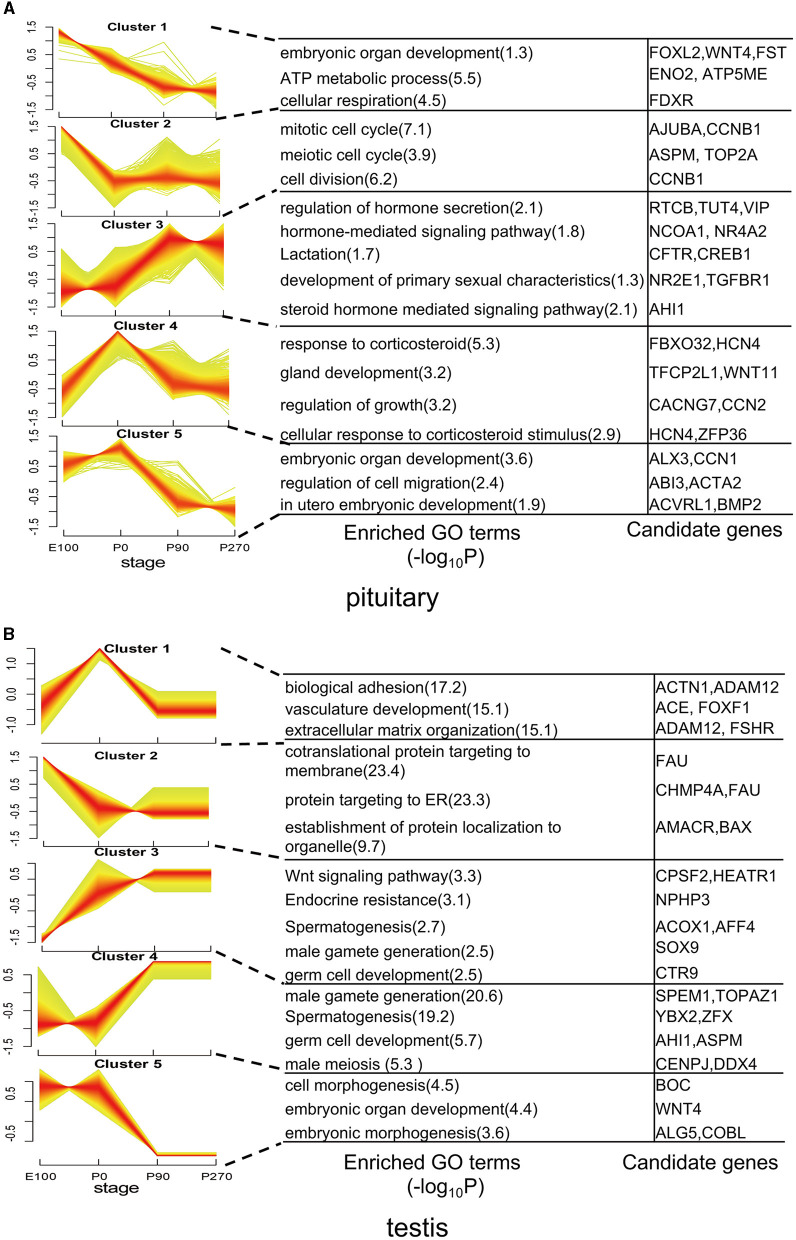
Dynamic expression patterns of genes during pituitary and testis development. **(A)** The clusters of differentially expressed genes in the pituitary. Corresponding biological processes are shown next to each cluster. **(B)** Similar to **(A)**, but for the testis.

To analyze the variation trend of differential genes, we classified all differentially expressed genes into five clusters based on their expression levels. In the pituitary, cluster 1 genes demonstrated a gradual decrease in expression levels across developmental stages, with significant (*P* < 0.01) enrichment in embryonic organ development and ATP metabolic processes. Conversely, Cluster 3 genes exhibited a progressive increase in expression levels across developmental stages and were significantly enriched in hormone secretion. Cluster 4 genes showed the highest expression levels at P0 and were significantly (*P* < 0.01) enriched in the regulation of growth (Figure 4A).

In the testis, genes in Cluster 1 exhibited elevated expression levels primarily during the early stages of development, particularly at birth. Notably, genes such as ACTN1, ACE, and FSHR within this cluster were significantly enriched in pathways related to biological adhesion and vasculature development. Conversely, Clusters 2 and 3 displayed contrasting expression patterns, while Clusters 4 and 5 exhibited opposing trends in gene expression. Additionally, these clusters differed in the enriched functional pathways, underscoring their distinct roles in testicular physiology (Figure 4B).

### 3.4 WGCNA and stage-specific gene reveal hup genes associated with different stages

**Figure 5:**
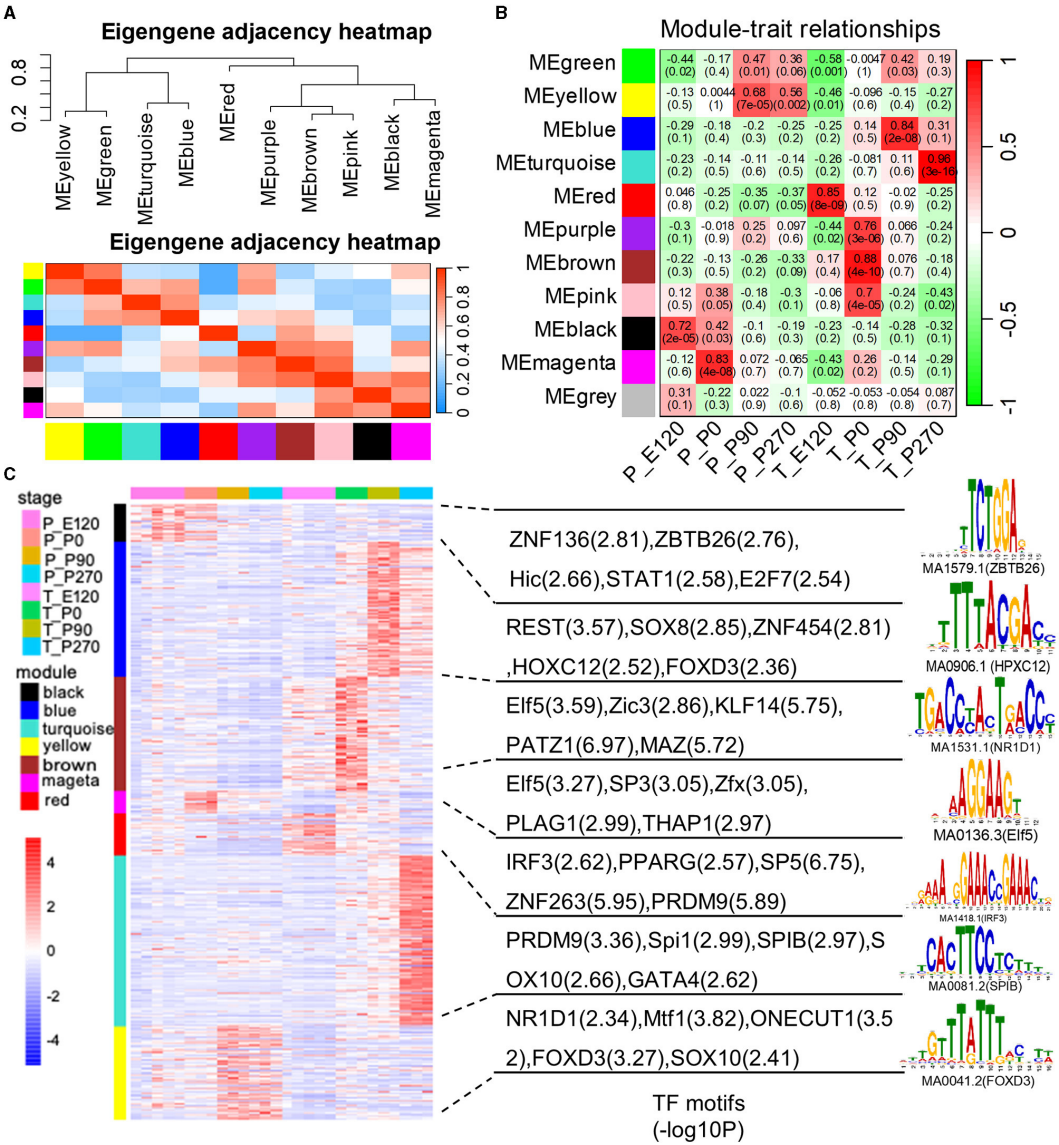
The weighted gene co-expression network analysis (WGCNA). **(A)** The eigengene dendrogram and heat map identify groups of correlated eigengenes cross modules. **(B)** Correlations between gene modules and developmental stages. The model-developmental stage relationship's statistical significance is corrected for multiple testing using the FDR method. The yellow stars denote FDR < 0.05. Each cell contains the correlation and the corresponding FDR value in the bracket. **(C)** Heatmap shows the normalized gene expression for genes in the top seven significant modules. The normalized gene expression for module-enriched TFs and the top representative sequence motif are shown next to each module.

We created gene co-expression networks for different developmental stages and identified eleven modules using the weighted gene co-expression network analysis (WGCNA) (Figure 5A). We then analyzed the correlation between each module and the different stages of tissue development (Figure 5B). To determine whether certain transcription factors (TFs) were regulating the genes in these stage-specific modules, we performed a motif enrichment analysis on the promoters of stage-specific genes separately (Figure 5C). We found that these TFs also exhibited stage-specific expression, indicating that they might play crucial roles in embryo and organ development.

**Figure 6:**
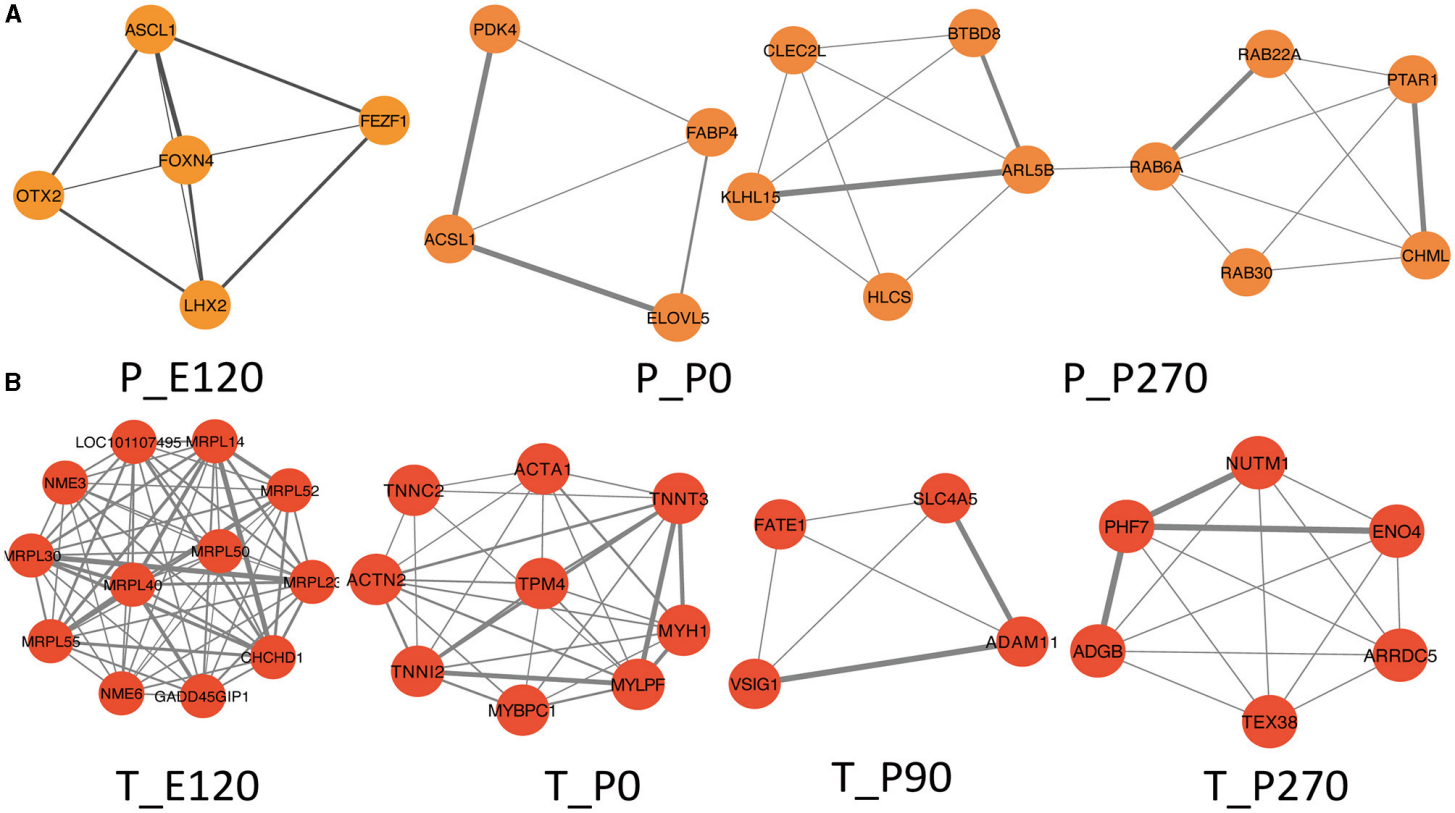
Hup network construction among different stages. **(A)** Subnetworks screened by MCODE among different stages in pituitary, MCODE score = 4.5, 3.3, 4.2. **(B)** Subnetworks screened by MCODE among different stages in testis, MCODE score = 12, 8, 5, 7.

Next, we generated Venn diagrams to intersect genes from stage-specific modules with those upregulated at each developmental stage. Through KEGG functional enrichment analyses, we observed a predominant enrichment of these genes in reproductive signaling pathways, including the cAMP signaling pathway, TNF signaling pathway, and Notch signaling pathway (Supplementary Figure S2). Subsequently, we employed these genes to construct a Protein-Protein Interaction (PPI) network. The MCODE algorithm was applied to extract pivotal subnetworks, identifying them as hub modules. For example, in the pituitary at E120, a network was identified among ASCL1, OTX2, FOXN4, LHX2, and FEZF1. In the P0 group, a network comprising ACSL1, ELOVL5, FABP4, and PDK4, was established. In the P270 group, networks were constructed among genes like PTAR1, CHML, ARL5B, and others. However, for the P90 group, no interaction network was established due to minimal interactions (Figure 6A). In the testis, we constructed four stage-specific modules. The E120 group module comprised twelve genes, including MRPL30 and NME3. The P0 group module consisted of fifteen genes, including TPM4 and MYH1. The P90 group module included four genes, such as FATE1, SLC4A5, VSIG1, and ADAM11. The P270 group module comprised six genes, including NUTM1, PHF7, ADGB, and ENO4 (Figure 6B). Importantly, the genes within these subnetworks demonstrated significant correlations with each other, suggesting a tightly regulated network of interactions that are critical at each developmental stage.

### 3.5 Exploration of pituitary and testis cell-cell interaction patterns

We analyzed the single-cell RNA sequencing data to explore interactions between the pituitary and testis. After rigorous processing and filtration of raw data, 23,837 cells in total were identified, among which, 10,927 testis cells and 12,910 pituitary cells were used for further analysis. Cell clustering revealed a clear separation between pituitary cell clusters and testis cell clusters (Supplementary Figure S3A), indicating the high quality of the data used for analysis. After conducting more analysis, it was discovered that there are eight different types of pituitary cells and 13 different types of testicular cells (Supplementary Figure S3B).

**Figure 7:**
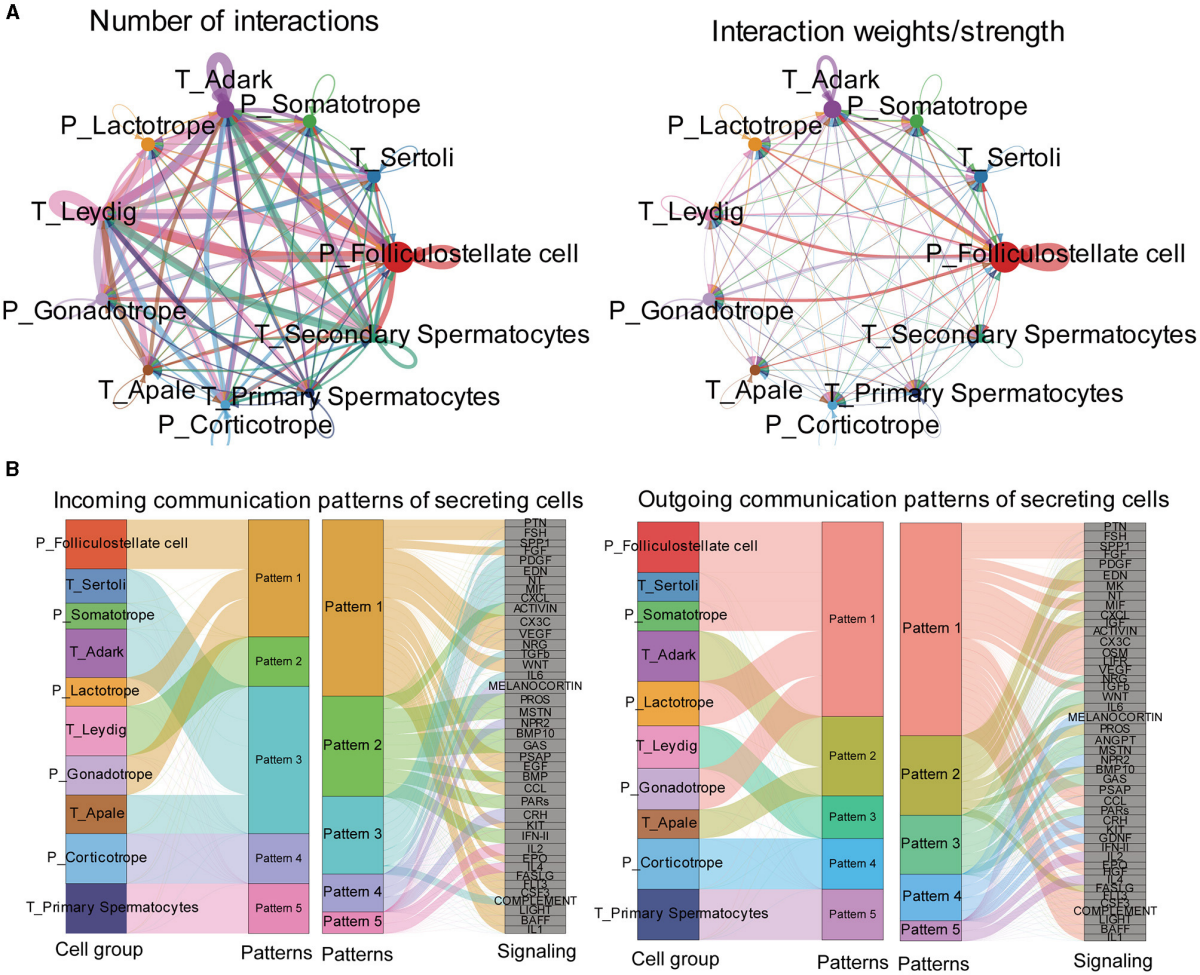
Exploration of the pituitary and testis cell-cell interaction patterns. **(A)** Circle plot of the ligand-receptor pairs of all cell types, with the thickness of the string representing the number of ligand-receptor pairs. **(B)** The river plot shows the contribution of ligands and receptors to different patterns and the contribution of patterns to different cell types.

Then, we predicted major signaling inputs and outputs for different cell clusters. We identified a list of ligand-receptor pairs among the cell clusters, such as TGFβ, FSH, PTN, BMP, NPR2, HGF, and IGF pathways. The numbers and weights of the ligand–receptor pairs were calculated and detailed (Figure 7A). Among all cell types, folliculostellate cells, gonadotrope cells, adark, Leydig cells, and somatotropes exhibited relatively high activity. We analyzed cell communication patterns among various cell types, considering the similarity in ligand and receptor expression (Figure 7B). Our observation indicated that the ligand patterns of secreting cells automatically clustered based on similar cell types.

### 3.6 BMP, HGF, IGF, and TGFβ signaling network among all cell types

**Figure 8:**
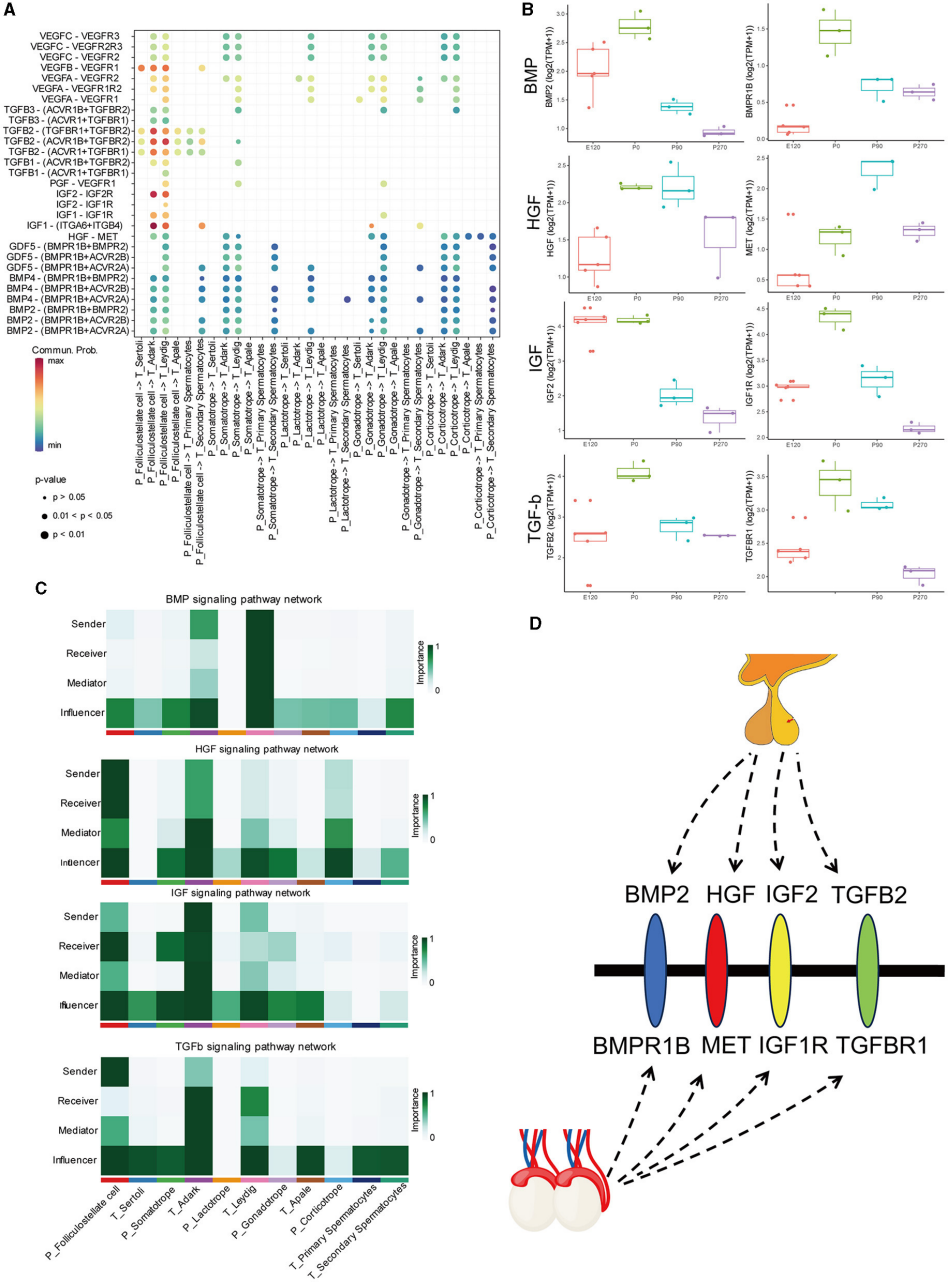
BMP, HGF, IGF, and TGFβ signaling network among all cell types. **(A)** Bubble plot of the ligand-receptor pairs of some cell types. **(B)** The boxplot shows the expression of ligands and receptors in BMP, HGF, IGF, and TGFβ signaling pathways across different stages of the pituitary and testis. **(C)** Heatmap shows the relative importance of each cell group based on the computed four network centrality measures of BMP, HGF, IGF, and TGFβ signaling network, respectively. **(D)** Hypothesis of the pituitary–testis axis.

To investigate the regulatory networks between the pituitary and testis at various developmental stages, we conducted signal pathways analysis that influence testicular regulation from the pituitary (Figure 8A). Our focus was on signal pathways including BMP, HGF, IGF, and TGFβ, for which we computed the communication probability between each pair of cell types involved. Our findings indicated that the BMP signaling pathway predominantly functions within Leydig cells of the testis. The HGF signaling network exhibited maximal active among folliculostellate cells, dark Leydig cells, and corticotrope. For the IGF signaling network, high activity was observed among folliculostellate cells, adark cells, and somatotrope cells. In the TGFβ signaling pathway, folliculostellate cells in the pituitary mainly act as senders, while adark cells in the testis predominantly function as receivers and other cells act as influencers in this pathway (Figure 8B).

Additionally, we analyzed the expression patterns of key ligands and receptor genes within these signaling pathways in the pituitary and testis across different stages. We observed that the expression trends of these ligand and receptor genes were largely consistent in both the pituitary and testis. Specifically, in the HGF signaling pathway, the gene expression of the ligand HGF showed relatively higher expression in the pituitary at birth and 90 days compared to other stages. In contrast, its receptor gene MET exhibits the highest expression in the testis at 90 days. Similarly, in the TGF signaling pathway, the ligand gene TGFB2 and the receptor gene TGFBR1 exhibited high expression levels in the pituitary and testis at birth, respectively (Figure 8B).

## 4 Discussion

The HPT axis plays an important role in reproduction, with extensive research focusing on the hypothalamus (18, 19), pituitary (20), and testis (21) across various developmental stages. The pituitary gland is critical in mammalian reproduction and growth development by secreting hormones such as GH, FSH, LH, and PRL. Notably, endocrine cells appear on the gestation of 60 days in the pituitary of sheep (22). A study identified FSHβ cells in the fetal pituitary at day 100, accompanied by numbers increasing as the gland matures (23). In our study, we explored pituitary development in sheep at 120 days of the embryonic and the other three postnatal stages using RNA-seq. We identified 380, 242, 34, and 479 specific-stage genes in the pituitary among the four developmental stages, respectively. Transcriptome dynamics primarily regulates testis development and spermatogenesis in a dynamic and stage-specific manner (24). The cell types in the testis differ at various stages of development through morphological analysis. Consistent with this observation, 886, 297, 201, and 3,678 specific-stage genes were identified in the testis. During their development, ram testes undergo three histological changes: prepuberty, maturity, and adulthood. During the stage of pre-puberty, which lasts from the first month until the fifth month, the process of spermatogenesis does not begin. These results are consistent with those observed by histologic morphology. The stage of spermatogenesis begins between 6 and 9 months of age, when the production of spermatozoa is initiated (25). Notably, the increased number of genes identified at the P270 stage suggests a potential link with spermatogenesis and sexual maturation.

The development of pituitary and testis is regulated by core genes (26). In this study, we utilized various analytical methods to construct the specific-gene network at each stage. In the pituitary, most of the stages-specific genes were enriched in embryonic organ development and mitotic cell cycle in the E120 and P0 groups. And the specific-gene network mainly contained OTX2, ASCL1, FOXN4, and other genes. Previous studies have suggested that these genes play a crucial role in both pituitary development and stem cell differentiation (27, 28). A single-cell RNA sequencing analysis of the developing human pituitary gland revealed that ASCL1 expression was a prominent feature of Pro.PIT1_all cells (29). It indicates that the pituitary glands of embryos and newborns possess stem cell properties and play a vital role in regulating the proliferation and differentiation of endocrine cells. Besides, we identified key genes in the pituitary of P90 and P270, but only the genes in P270 conducted a network. The main functions of these genes are vesicle-mediated transport and metabolism of proteins. This may be related to pituitary hormone secretion. In the embryonic stage, mitochondrial ribosomal protein genes are the core genes, indicating that energy metabolism mainly occurs in the testis. In the newborn group, the core network primarily comprised genes associated with muscle structure (30). At P90 and P270 days, the core network mainly consists spermatogenesis-related genes. For example, *VSIG1*, a member of the junctional adhesion molecule family, is primarily expressed in the stomach and testis, and plays important roles during spermatogenesis (31). Earlier studies have highlighted ENO4 as the primary enolase in mouse sperm, and its absence leads to structural abnormalities in sperm (32). This underscores the absence of mature sperm in the testicles at 90 days, with spermatogenic functions predominating. By P270 days, mature sperm are evident in the testis, with a majority of key genes participating in the regulation of sperm fertilization. During the development of the pituitary and testis, key genes play a crucial role in the production of sperm.

Spermatogenesis is regulated by the coordinated endocrine action of the HPT axis. GnRH secreted by the hypothalamus is transported to the anterior pituitary, which stimulates the gonadotrophin to secrete LH and FSH. LH stimulates Leydig cells to produce testosterone, while FSH regulates spermatogenesis via its effects on Sertoli cells in the seminiferous tubules (33). Numerous other signaling pathways play a crucial role in regulating this process. Therefore, we used single-cell RNA sequencing data from the pituitary and testis to investigate the signaling pathways that act on the testis and identify potential underlying mechanisms. The signaling pathways, such as BMP, HGF, IGF, and TGFβ were highly active in the pituitary and testis cell types. Previous experiments in mouse testis have shown that BMPR-1B signaling inhibits testosterone production by regulating HSD isoforms and aromatase, both *in vivo* and *in vitro* (34). It has been demonstrated in previous studies that bone morphogenetic proteins (BMPs) play a crucial role in male reproduction, along with LH and FSH (35–37). In the present study, it was discovered that the pituitary gonadotrope is linked to spermatocytes in the testis through the BMP pathway. The HGF signaling pathway is actively expressed at all stages of testicular development (38, 39). This was confirmed by the expression of MET in this experiment. Besides, HGF can stimulate protease secretion in stromal cells, enhance TGF-β activity, modulate tight junctions between supporting cells, and impact the formation of the blood-testis barrier (40). *In vitro*, cultured testicular stromal cells were observed to synthesize testosterone, develop embryonic testicular stromal cells, and increase testicular stromal cell survival when stimulated by HGF (41). A recent report highlighted the significance of IGFs as the primary growth factor in regulating the number of SCs and testis size (42). SCs are the sole somatic cells present in the seminiferous epithelium and play a crucial role in supporting spermatogenesis (43). *In vivo* experiments have demonstrated that the proliferative effects of FSH on immature SCs during the neonatal stage are mediated through the insulin/IGF signaling pathway. This indicates the involvement of insulin/IGF signaling in facilitating the actions of FSH on SC proliferation (42). TGF-β superfamily ligands regulate testis development by controlling germline fate specification and cellular reorganization that underlies testis formation in embryos. Besides it can influence both somatic and germ cells during the onset of spermatogenesis in juvenile testis growth (44). Activins and inhibins in TGFβ pathways play a key role in regulating the hypothalamic-pituitary-gonadal axis in domestic animals. To summarize, the pituitary-testicular axis is significantly influenced by these four signaling pathways.

## 5 Conclusion

In summary, we characterized the global changes of the transcriptome across four developmental stages of the pituitary and testis in sheep. Besides, according to scRNA data, we provided fundamental evidence that the pituitary might regulate testis functions through multiple signaling pathways, the mechanism associated with our findings should be validated in the future.

## Data availability statement

The original contributions presented in the study are publicly available. This data can be found here: https://www.ncbi.nlm.nih.gov/bioproject/; PRJNA1066045.

## Ethics statement

The animal study was approved by Animal Care and Use Committee of Nanjing Agricultural University. The study was conducted in accordance with the local legislation and institutional requirements.

## Author contributions

SL: Conceptualization, Investigation, Writing—original draft, Writing—review & editing. BZ: Data curation, Formal analysis, Software, Writing—review & editing. HY: Data curation, Investigation, Writing—review & editing. KD: Data curation, Investigation, Writing—review & editing. YC: Writing—review & editing. HX: Writing—review & editing. CP: Data curation, Investigation, Writing—review & editing. FW: Funding acquisition, Supervision, Writing—review & editing. YZ: Conceptualization, Funding acquisition, Project administration, Supervision, Writing—review & editing.
