# Supplementary material for: Comprehensive transcriptomic analysis revealing the regulatory dynamics and networks of the pituitary-testis axis in sheep across developmental stages
Source: Front Vet Sci. 2024 Feb 19;11:1367730. doi: 10.3389/fvets.2024.1367730 (PMC10909840; doi:10.3389/fvets.2024.1367730)
Supplement: Supplementary file 1 [file Data_Sheet_1.docx]

**Supplemental Materials**

**
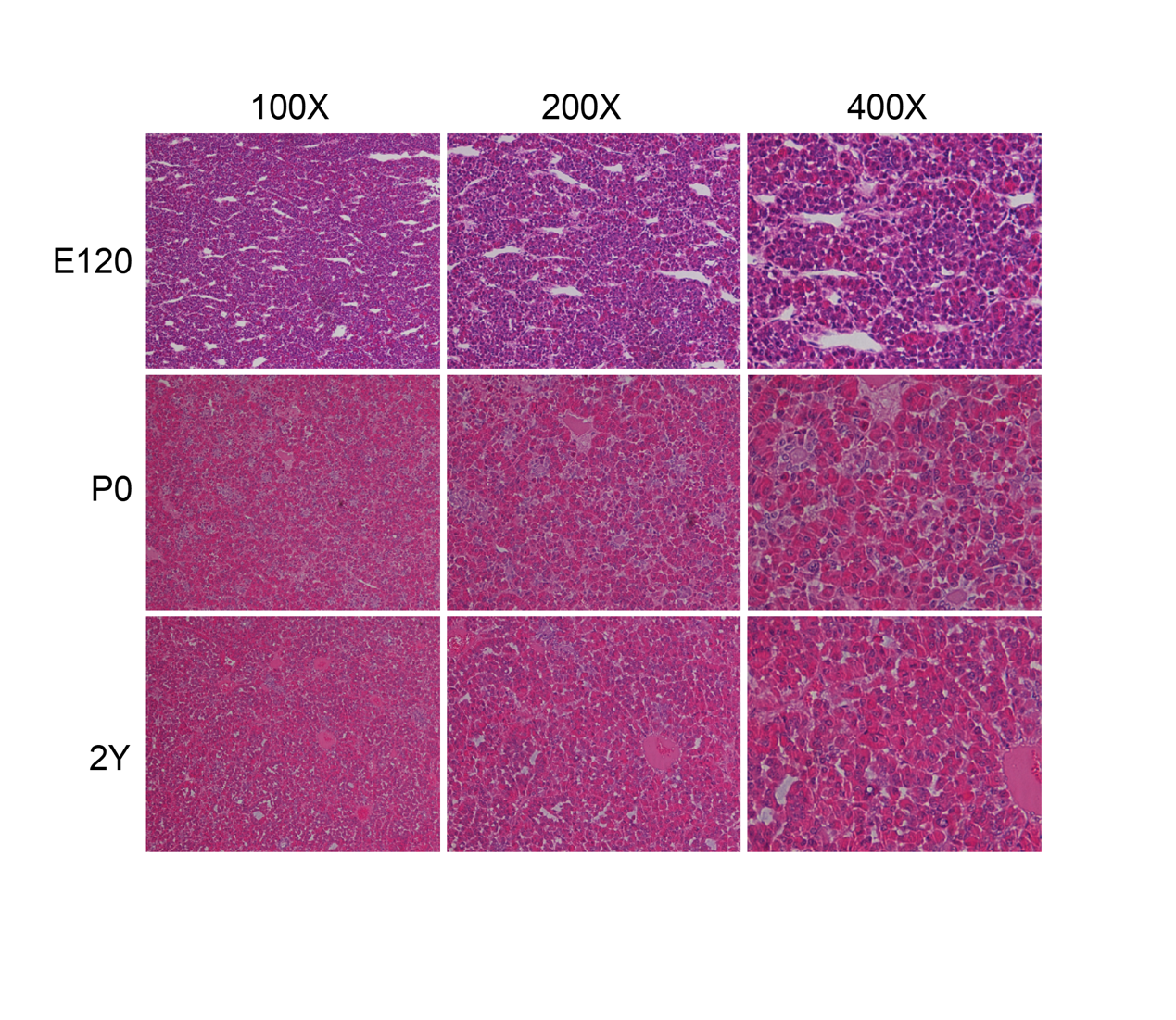
**

**Fig. S1 Histological analysis of the pituitary at different developmental stages.**

The pituitary tissues were observed under the microscope at ×100, ×200, and ×400 magnification.


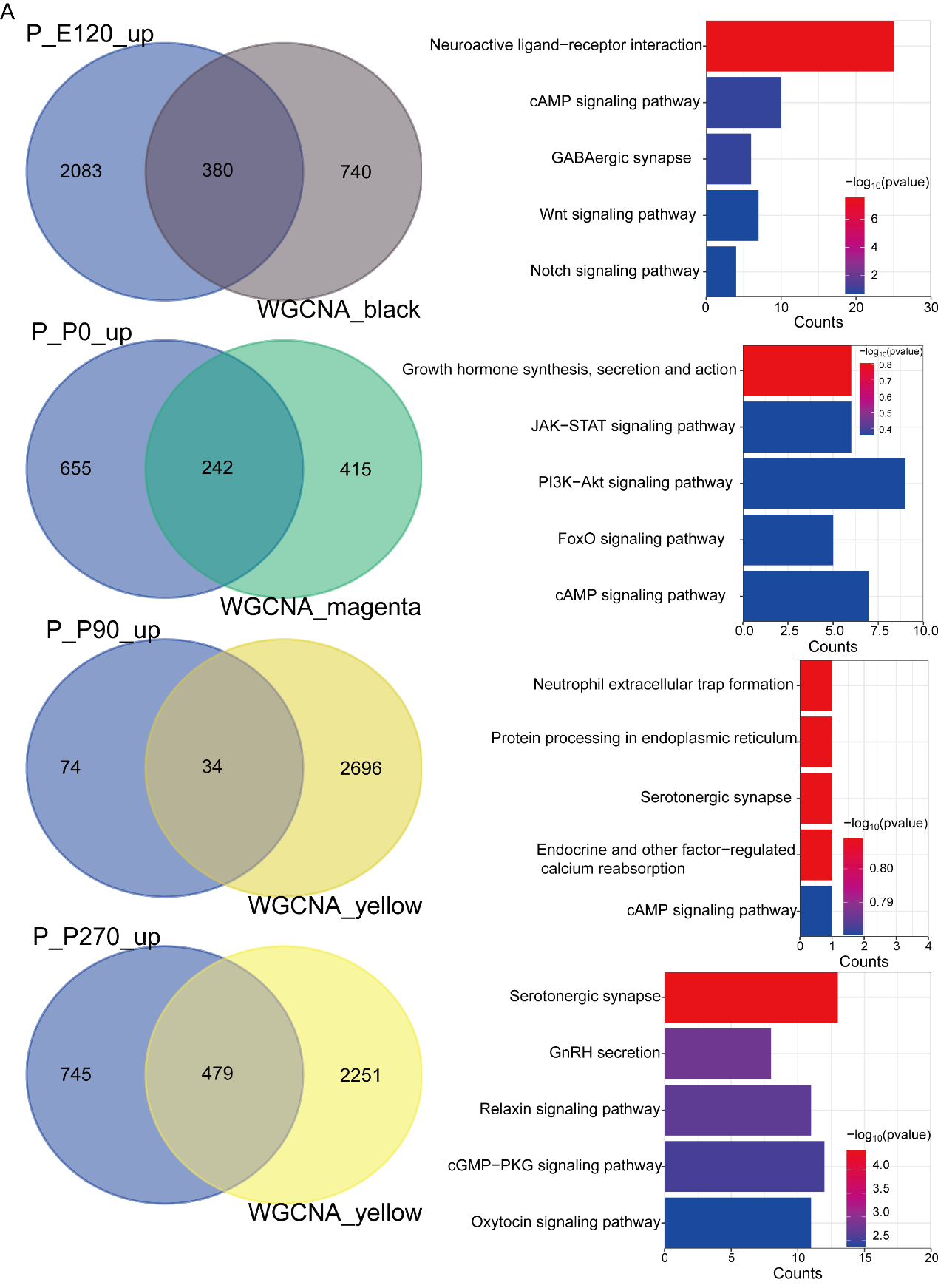


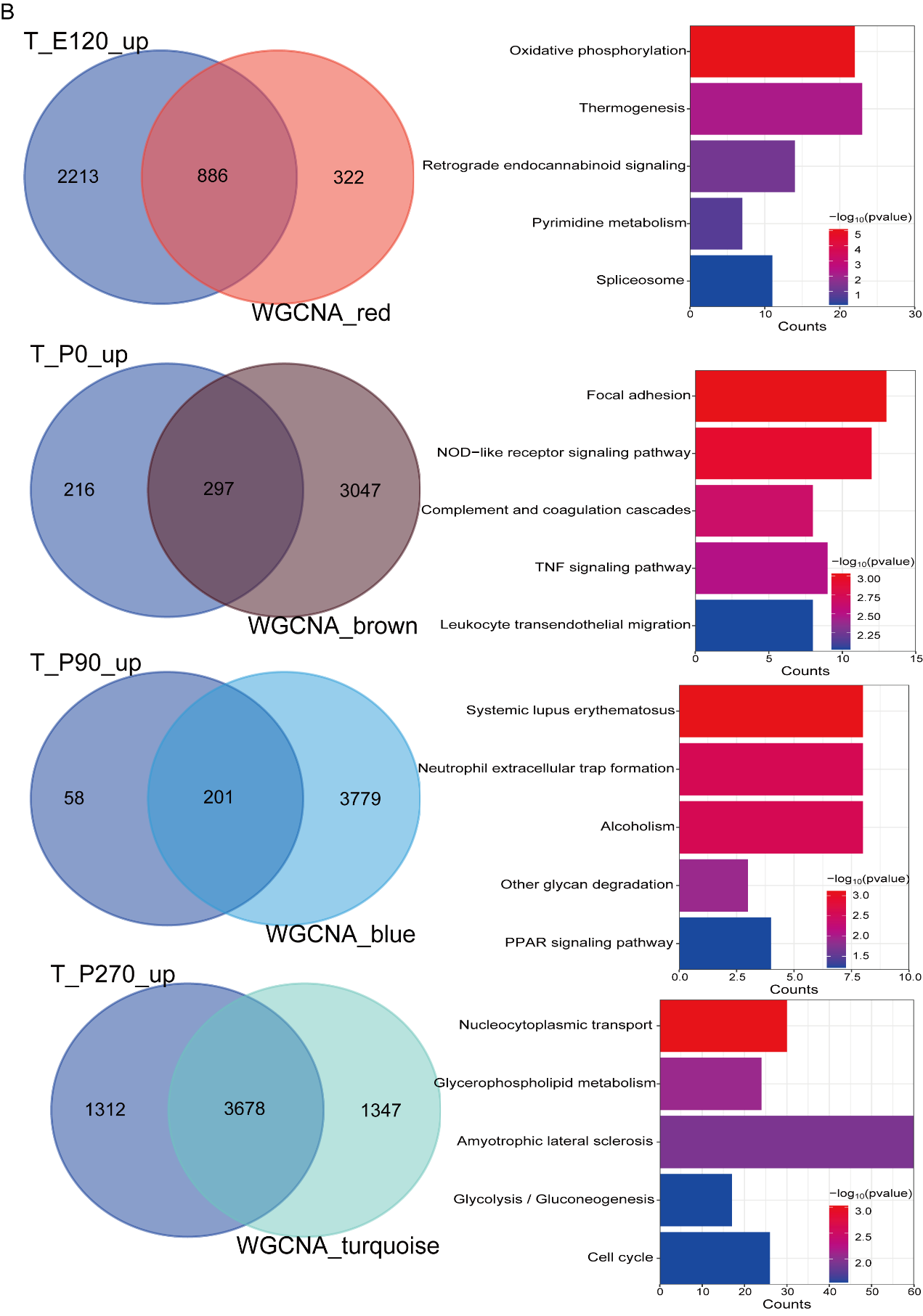


**Fig. S2 Venn diagram showing key genes**

**A** The Venn diagram(left) illustrates the overlap between genes upregulated at different stages and genes within WGCNA-related modules in the pituitary. On the right, the associated genes enrich the KEGG pathway.

**B** Similar to A, but for the testis.

**
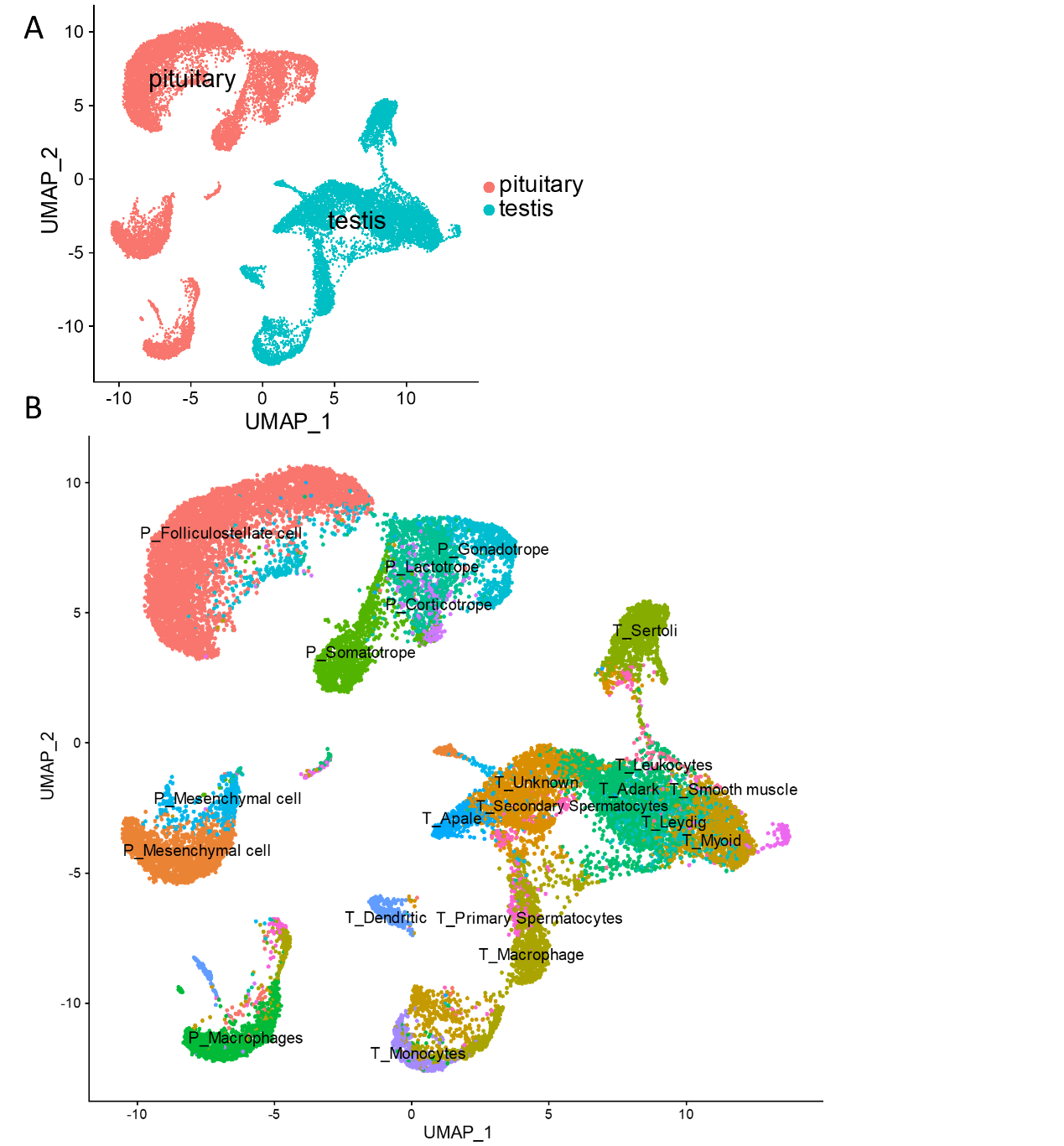
**

**Fig. S3 Combination of the pituitary and testis single-cell transcriptomic data.**

**A** Uniform Manifold Approximation and Projection (UMAP) plot of the sheep pituitary and testis. **B** UMAP shows twenty-one different cell cluster annotations of the combined pituitary and testis single-cell transcriptomic data.
